# Effect of heart rate control with amiodarone infusion on hemodynamic and clinical outcomes in septic shock patients with tachycardia: a prospective, single-arm clinical study

**DOI:** 10.1186/s40780-021-00219-6

**Published:** 2021-10-11

**Authors:** Masoud Khataminia, Farhad Najmeddin, Atabak Najafi, Hamidreza Sharifnia, Arezoo Ahmadi, Adeleh Sahebnasagh, Mojtaba Mojtahedzadeh

**Affiliations:** 1 Student Research Committee, Faculty of Pharmacy, Tehran University of Medical Sciences, Tehran, Iran; 2 Department of Clinical Pharmacy, Faculty of Pharmacy, Tehran University of Medical Sciences, Tehran, Iran; 3 Department of Anesthesiology and Critical Care Medicine, Sina Hospital, Tehran University of Medical Sciences, Tehran, Iran; 4 Department of Anesthesiology and Critical Care, Sina Hospital, Tehran University of Medical Sciences, Tehran, Iran; 5 Department of Anesthesiology and Critical Care Medicine, Sina Hospital, Tehran University of Medical Sciences, Tehran, Iran; 6 Department of Internal Medicine, Clinical Research Center, North Khorasan University of Medical Sciences, Bojnurd, Iran; 7 Department of Clinical Pharmacy, The Institute of Pharmaceutical Sciences (TIPS), Tehran University of Medical Sciences, Tehran, Iran

**Keywords:** sepsis-induced tachycardia, Amiodarone, Complications, Pelvic neoplasms, Prevention

## Abstract

**Background:**

Keeping the heart rate within the normal range has improved the survival of septic shock patients. Amiodarone could target the underlying pathophysiology of sepsis-induced tachycardia. This study aimed to determine whether amiodarone is effective in controlling the heart rate in critically ill patients with septic shock and sustained tachycardia who were receiving vasopressor.

**Methods:**

In this prospective, single-arm cohort study, 46 patients with septic shock and tachycardia were enrolled to receive a loading dose of amiodarone 150 mg, then continuous infusion of 1 mg/min. The primary outcome was the ability of amiodarone in rate control lower than 95 beats per minute (BPM) and maintaining it during 24-h study period. We also recorded the effect of amiodarone on hemodynamic indices as the secondary outcomes.

**Results:**

The results of the present study indicated a significant decrease in HR in septic shock patients for amiodarone, from 121.0 (116.5, 140.0) at baseline to 91.5(89.3, 108.0) at the end of the study period (*p* < 0.001). During the study period, a total of 26 (56.52%) of patients achieved the target heart rate lower than 95 BPM and maintained it during study period. Amiodarone decreased HR by 22.8 ± 13.7. While receiving amiodarone infusion, the values for heart rate, mean arterial pressure, cardiac index, norepinephrine infusion rate, and stroke volume index changed significantly between amiodarone initiation and 24-h follow-up (*P* < 0.001). Amiodarone was well tolerated, because this anti-arrhythmic agent did not increase the need for vasopressor and none of the patients experienced episodes of refractory hypotension.

**Conclusion:**

This study showed that amiodarone infusion successfully reduced the heart rate in sepsis-induced tachycardia. The patients had improved hemodynamic state as indicated by an increase in cardiac index and SVI.

## Background

Sepsis and septic shock are the result of exaggerated immune responses to infection, which could promote to fatal organ dysfunction. Septic shock is the cause of around 30% mortality in the intensive care unit (ICU) and the primary cause of mortality in 58.3% of patients being discharging from ICU [1, 2]. Following the septic shock, the immune system provokes immense responses to the invading pathogens. This phenomenon persuades an overwhelming release of catecholamines, which accelerates the following pathological events: sustained tachycardia, cytosolic calcium overload, cardiac stiffness, shortened diastolic phase, decreases in stroke volume (SV) and ejection fraction in the late stages of sepsis [3, 4]. Furthermore, the capillary leak may occur as a result of decreased arteriolar resistance and low systemic vascular resistance (SVR), which poorly response to exogenous catecholamines. These extensive and elaborated immune responses consequently result in multiple organ dysfunction [5, 6].

Generally, the septic shock patients suffer from tachycardia, even after modifying other etiologies of tachycardia, including hypovolemia, anemia, pain and agitation [7]. Sepsis-induced tachycardia independently correlates with the patient’s clinical outcome. The possible explanation for this poor consequence is the increased myocardial workload and oxygen demand following tachycardia [8]. On the other hand, keeping the heart rate within the normal range has improved the survival of septic shock patients [9]. The underlying pathophysiology of sepsis-induced tachycardia is calcium dysregulation and excessive adrenergic responses [10, 11]. Under physiological conditions, originated calcium from the sarcoplasmic reticulum is responsible for cardiomyocyte contractility. In sepsis, the sensitivity of cardiomyocyte to the calcium slackens off and the binding of calcium to troponin is attenuated. The responsiveness of the ryanodine receptor to calcium is also diminished [12]. Moreover, the expression of calpain increases in cardiac cells during overwhelming inflammation [13]. Calpain enzymes are cytosolic cysteine proteases, that they are activated by calcium. Calpain overstimulation consequently results in myocardial remodeling and heart failure. Since this enzyme has destructive effects, the inhibition of calpain could potentially be beneficial in sepsis-induced cardiomyopathy [14].

Similarly, overwhelming adrenergic responses also contribute to tachycardia, myocardial suppression, thrombogenicity, impaired immune system and invading pathogens overgrowth. Furthermore, the high plasma catecholamines correlate with poor hemodynamic and neurological outcomes [11]. Circulating catecholamines have adverse effects on the heart via oxidative stress and cellular apoptosis in myocardiocytes [15]. The results of some clinical studies suggested that blockade of β-receptors has some beneficial effects in septic patients with persistent tachycardia by presiding over the sympathetic overflow [16, 17]. In septic shock patients receiving vasopressor, Esmolol infusion is associated with improved hemodynamic and clinical outcomes. Furthermore, β-receptors blockade reduces oxygen demand, improves coronary perfusion and stabilizes the hemodynamic state [18].

Amiodarone is a well-known rate-controlling cardiac dysrhythmia medication, which can block both β –receptors and calcium channels concomitantly [19]. This unique agent has also shown neuroprotective effects via blockade of the sodium channels in ischemic brain injury models [20]. Thereby, it is presumed that administration of amiodarone with both β-receptor and calcium channel blockage better targets the underlying pathophysiology of sepsis-induced tachycardia and enhances the outcomes of the septic patients with tachycardia. This is through extending the diastolic phase, enhancing the cardiac function and correcting the calcium dysregulation. Unlike β-blockers, there is no concern about amiodarone causing hypotension or deterioration of cardiovascular function [21]. Moreover, amiodarone is well tolerated with no adverse effects on hemodynamic parameters, including mean arterial blood pressure (MAP) and cardiac output (CO) with a better tolerability profile [22].

Therefore, the present study aimed to determine whether amiodarone is effective in controlling the heart rate in critically ill patients with the septic shock and sustained tachycardia who were receiving vasopressor. This is the first clinical study of amiodarone for controlling the heart rate in the septic shock. In addition to heart rate control as our primary endpoint, we evaluated the secondary endpoints of the increased vasopressor demand, metabolic parameters (lactate concentration) and also deterioration of hemodynamic indices, including Stroke Volume Index (SVI), SVR, MAP, and cardiac index (CI) over time.

## Methods

### Patients

This is a prospective, single-arm, preliminary study, evaluating the efficacy of amiodarone in rate control in the septic shock patients with tachycardia. This study was approved by the Tehran University of Medical Sciences ethics committee (Ethics code: IR.TUMS.VCR.REC.1398.426) and carried out in two multidisciplinary ICU) affiliated to Tehran University of Medical Sciences (Tehran, Iran). We provided a written informed consent, which was signed by patient’s next of kin.

### Material

Amiodarone was purchased from Hameln Pharma Plus GmBH. We used USCOM (Ultrasonic Cardiac Output Monitors) to calculate and record hemodynamic indices (Australia).

### Study population

All the patients were mechanically ventilated and under sedation. The inclusion criteria were as follows: all the septic shock patients aging 18 years or more, with persistent tachycardia (heart rate > 95 beats/min) who were dependent on vasopressor to maintain a MAP of 65 mmHg or above, despite adequate volume resuscitation. The definition of shock is according to Surviving Sepsis Campaign update [23]. The management of sepsis was performed according to the Surviving Sepsis Campaign guideline [24], so that after resuscitation and initiation of vasopressor to maintain the MAP, as well as source control and taking samples from blood and the possible source of infection for microbial culture, broad-spectrum empirical antibiotic treatment was administered. We then modified antibiotic treatment based on the results of culture and response to the initial treatment. All patients received the same sedation protocol based on fentanyl and without midazolam. If the patients became restless, they received low doses of midazolam.

The exclusion criteria were as follows: the patients who received β-blocker or amiodarone in the last 48 h, patients receiving Extracorporeal membrane oxygenation (ECMO), therapeutic hypothermia or vasopressor other than Norepinephrine (NE), known history of amiodarone intolerance, Acute respiratory distress syndrome (ARDS) with arterial oxygen partial pressure to fractional inspired oxygen (PaO_2_/FiO_2_) less than 150, Atrial fibrillation (AF), previous use of β-blocker, digoxin or non-dihydropyridine calcium channel blocker in the last two weeks, history of heart valve diseases, cardiogenic shock (Cardiac index < 2.2 L/min/m^2^), history of severe heart failure (New York Heart Association Classification≥3), arrhythmias other than Atrial Fibrillation or Premature atrial/ventricular contraction more than two every 20 s, history of lung fibrosis and pregnancy.

### Measurement of hemodynamic parameters by USCOM

USCOM is a device for advanced hemodynamic monitoring for optimizing the fluid therapy and administration of vasopressors. For this purpose, the operator placed the USCOM transducer on a suprasternal notch (aortic valve). By measuring the flow through the aortic valve, the device can accurately measure hemodynamic parameters, including blood flow, stroke volume and cardiac output. USCOM was performed by two operators separately. If the difference between the measured parameters was more than 10%, a third person would perform USCOM and finally, the parameters agreed by all three operators would be reported.

### Study protocols

From December 2019 to November 2021, 46 patients enrolled in this study. We administered amiodarone infusion to the eligible patients. After recording baseline hemodynamic indices of the patients, CO and SVR, treatment with intravenous (iv), amiodarone was initiated.

Tachycardic patients, due to other reasons, were eliminated or modified before enrollment. For this purpose, we evaluated pain (Critical Care Pain Observation Tool), agitation (Richmond Agitation Sedation Scale), and volume status [assessed by flow time corrected (FTc). If the patient had painless agitation, midazolam 3 mg IV was administered, followed by 1 mg/hr. infusion. FTc, as a predictor of adequate fluid therapy, was measured by USCOM. In patients with FTc values less than 300 milliseconds, we performed an additional passive leg raising (PLR) maneuver at the patient’s bedside to certify if the patient was hypovolemic. After the PLR maneuver, we performed an additional USCOM. If CO increased more than 10%, the patient was considered hypovolemia, and we initiated volume resuscitation by crystalloid fluid before enrollment [19]. Moreover, we administered 200 mg/day iv hydrocortisone to those patients who did not become hemodynamically stable despite receiving adequate fluid resuscitation and vasopressor [20].

Treatment with amiodarone was initiated with a loading dose of 150 mg over 10 min, then continued at the infusion rate of 1 mg/min. In patients who exhibited a heart rate < 60 beats per minute (BPM), the infusion was temporarily withheld [25].

### Outcome

The primary prespecified outcome was rate control lower than 95 BPM and maintaining it during the study period by amiodarone. As our secondary outcomes, we recorded the effect of amiodarone on hemodynamic indices (MAP, CI, SVR and SVI).

### Clinical data

After recording the demographic data of patients at the initiation of the study, the following values were evaluated for the study participants at baseline and then 6, 12, and 24 h after randomization: hemodynamic and blood gas indices, lactate levels and the need for vasopressor. Other laboratory data were recorded at baseline and after 24 h only. We also evaluated sequential organ failure assessment (SOFA) score [26], The Glasgow Coma Scale (GCS) [27] score, pain based on Critical Care Pain Observation Tool [28] and the incidence of agitation and delirium during ICU stay according to Richmond Agitation Sedation Scale (RASS) [29]. Moreover, the changes in systolic and diastolic blood pressure, MAP, and HR were all written down at six-hour intervals during the study period. However, in one of our ICU centers, it was only possible to measure hemodynamic parameters baseline and at the end of 24 h. Therefore, we attributed the last observation carried forwards method for missing data to perform analysis and comparison between subjects [30].

To continuously monitor hemodynamic measurements (MAP, CI, SVR and SVI), a validated USCOM device was applied.

### Statistical analysis

Continuous data summarized by mean ± standard deviation (SD) and qualitative data by frequency (%). The normality of continuous data evaluated by the Kolmogorov-Smirnov test. Baseline and post-intervention outcomes compared by paired samples T-test or Wilcoxon’s signed-rank test. Results in each post-intervention time was summarized by Bonferroni 95% confidence intervals (CIs) through error bars. The significant probability of two-tail tests was considered less than 0.05. SPSS version 23 (IBM Corp) was used for statistical analysis.

## Results

### Patients

**Fig. 1:**
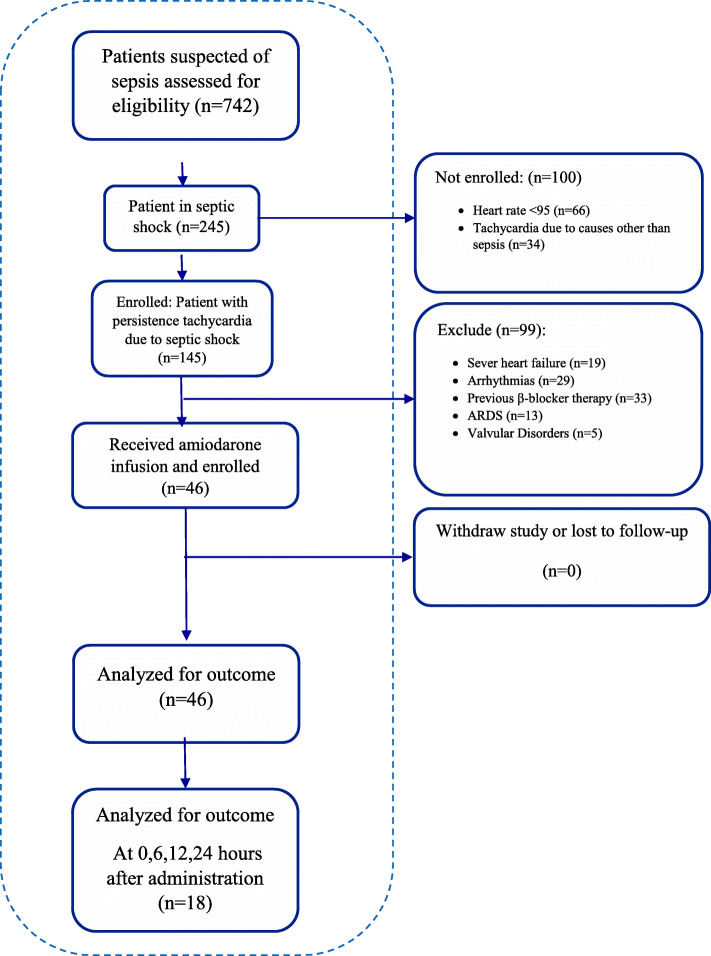
Flow diagram of participants

As illustrated in Fig. 1, from 742 patients suspected for sepsis, 145 patients were in the septic shock with sustained tachycardia after evaluation. A total of 99 patients excluded from the study for: history of severe heart failure, arrhythmias, previous β-blocker therapy, ARDS, and history of valvular disorders. After optimizing pharmacotherapy with sedation or fluid resuscitation, tachycardia resolved in 34 patients. In the end, a total of 46 patients consented and completed the study.

Baseline clinical characteristics of the patients displayed in Table 1. All the enrolled patients were mechanically ventilated and received sedation. In all patients, the only anti-arrhythmic medication to control heart rate was amiodarone. Moreover, all patients included in the study were in the septic shock based on Surviving Sepsis Campaign update [23]. Throughout the study, patients were closely monitored by the treatment team and the clinical investigator. The confounders of pain, agitation and delirium were corrected before patients’ enrollment and performance of USCOM. The mean age was 59.8 ± 11.3 years, and the mean SOFA score of the patients at the time of admission was 7.24 ± 1.8. Norepinephrine was infused at the mean rate of 10.7 ± 2.9 μg/min. None of the patients developed bradycardia or experienced adverse effects while receiving amiodarone infusion. In none of the patients, amiodarone infusion was held due to drug intolerance. The clinical research coordinator recorded iv fluid intake to achieve adequate volume expansion in patients during 24 h of the study. 17 out of 46 (37%) of patients needed fluid resuscitation during the study, as characterized by FTc values less than 300 milliseconds. None of the patients had overt volume overload.

**Table 1:** Baseline Characteristics of the Patients at the initiation of amiodarone infusion

Variable	Amiodarone treated patients (*n = 46*)
Age (year)	59.8 ± 11.3^a^
Sex (male)	23(50.0)^b^
BMI (kg/m2)	27.4 ± 3.2
SOFA SCORE	7.24 ± 1.8
PLT (10^3^/μl)	229.9 ± 85.1
BSA (m^2^)	1.93 ± 0.15
HR (bpm)	118.9 ± 18.7
MAP (mmHg)	98.4 ± 9.3
CI (l/min/m^2^)	3.61 ± 0.94
SVR (dynes/seconds/cm^−5^)	1106.8 ± 381.6
SVI (ml/m^2^/beat)	31.03 ± 8.5
NE (μ/min)	10.74 ± 2.9
FTC (milliseconds)	356.9 ± 78.6
LACTAT (mg/dL)	24.6 ± 11.5
**Comorbidities n (%)**	
Heart failure	10 (21%)
Diabetes mellitus	18 (39.1%)
Chronic kidney disease	13 (28.2%)
Previous MI	12 (26%)
Previous stroke	9 (19%)
Chronic obstructive pulmonary disease	1 (2.2%)
CRP	13.13 ± 10.59
GFR (mL/min/1.73 m^2^)	78.43 ± 16.56
Cirrhosis	3 (6.5%)

BMI: Body mass index, PLT: platelet, BSA: body surface area, HR: heart rate, MAP: mean arterial pressure, CI: cardiac index, SVR: Systemic vascular resistance, SVI: Stroke Volume Index, NE: Norepinephrine, FTC: flow time corrected, MI: myocardial infarction, CRP: c-reactive protein, GFR: Glomerular Filtration Rate

^a^ Mean ± standard deviation, ^b^ number (%)

During the study period, a total of 26 (56.52%) of patients achieved the target heart rate lower than 95 BPM and maintained it during study period. Patients received amiodarone infusion throughout the 24 h of the study. Amiodarone had a good safety profile and well tolerated, since amiodarone did not increase the need for the vasopressor throughout the study. None of the patients experienced episodes of refractory hypotension. None of the patients received vasopressin other than norepinephrine as vasopressor. On average, amiodarone decreased HR by 22.8 ± 13.7.

**Fig. 2:**
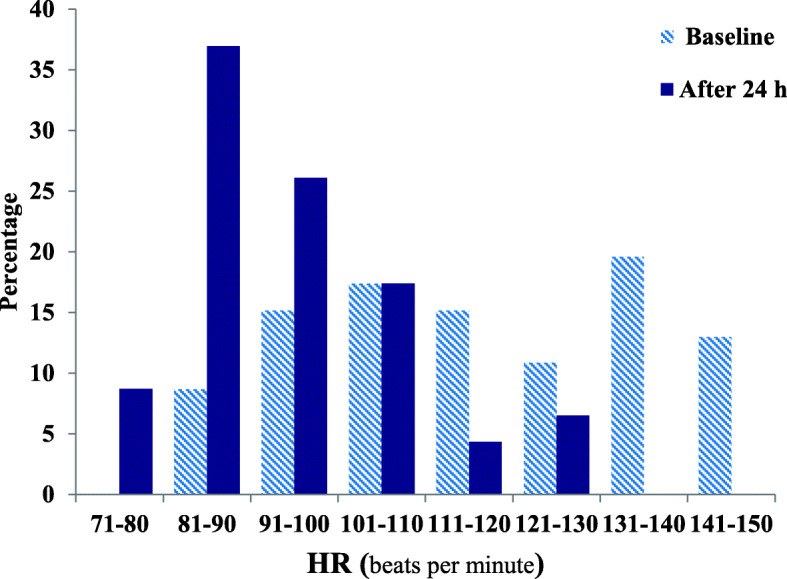
The frequency (%) of different ranges of heart rate in patients at enrolled and after study period

**Fig. 3:**
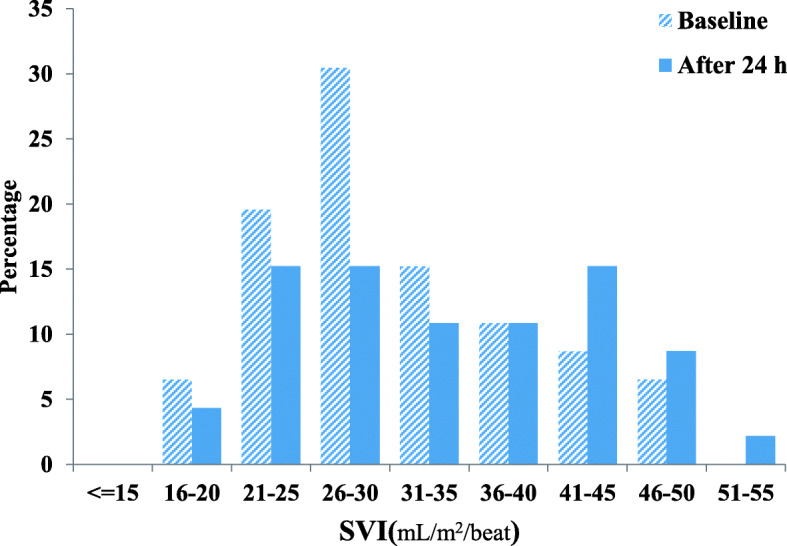
The frequency (%) of different ranges of SVI in patients at enrolled and after the study period

**Fig. 4:**
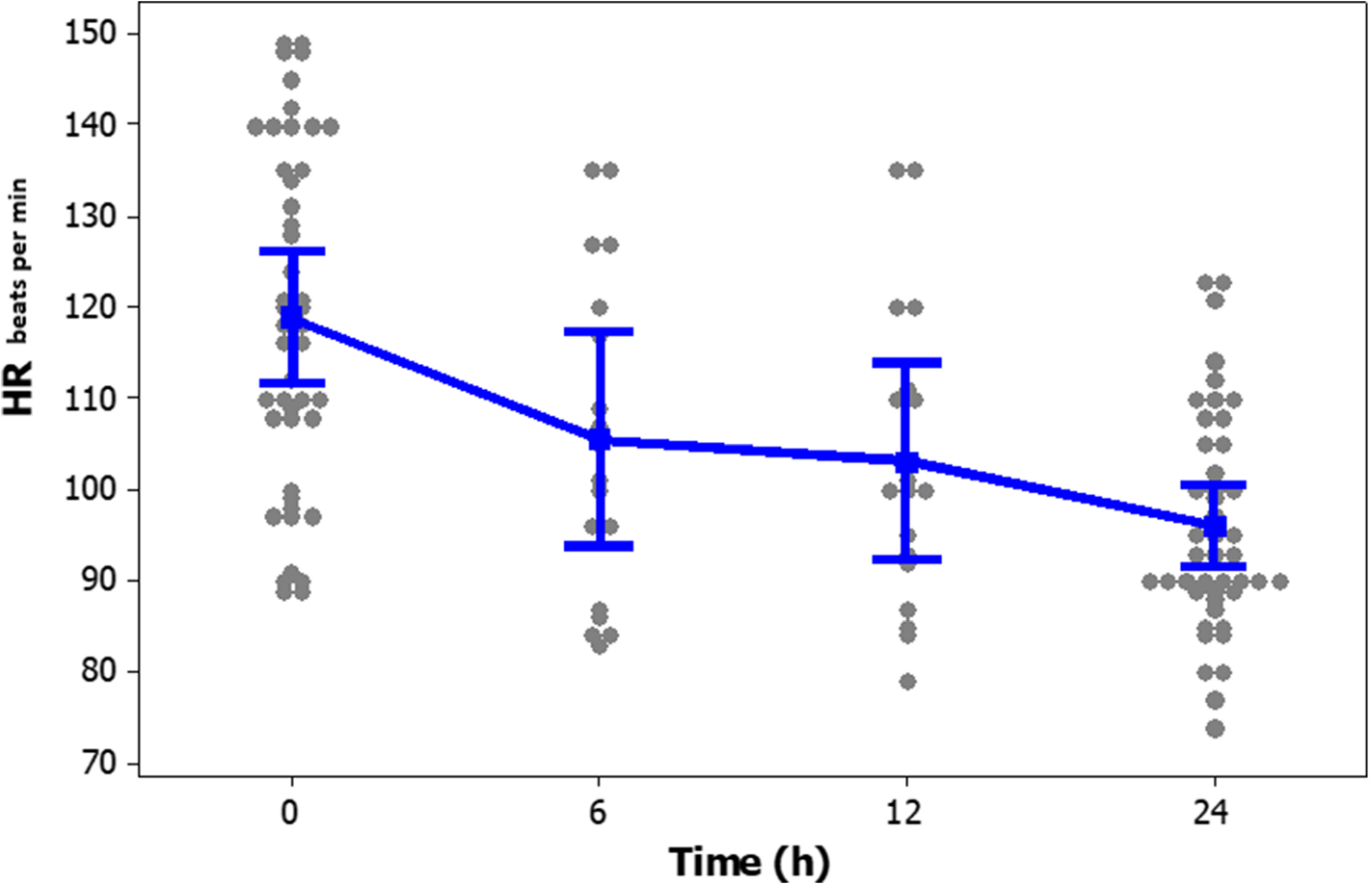
Mean and Bonferroni 95% CI of HR during 24-h of follow-up

**Fig. 5:**
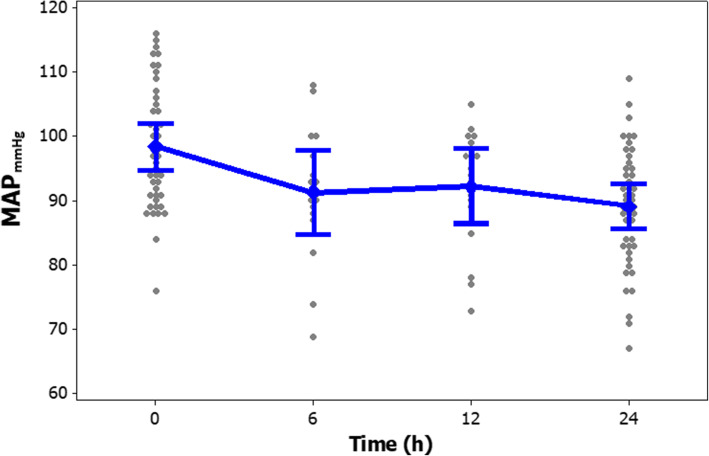
Mean and Bonferroni 95% CI of MAP during 24-h of follow-up

**Fig. 6:**
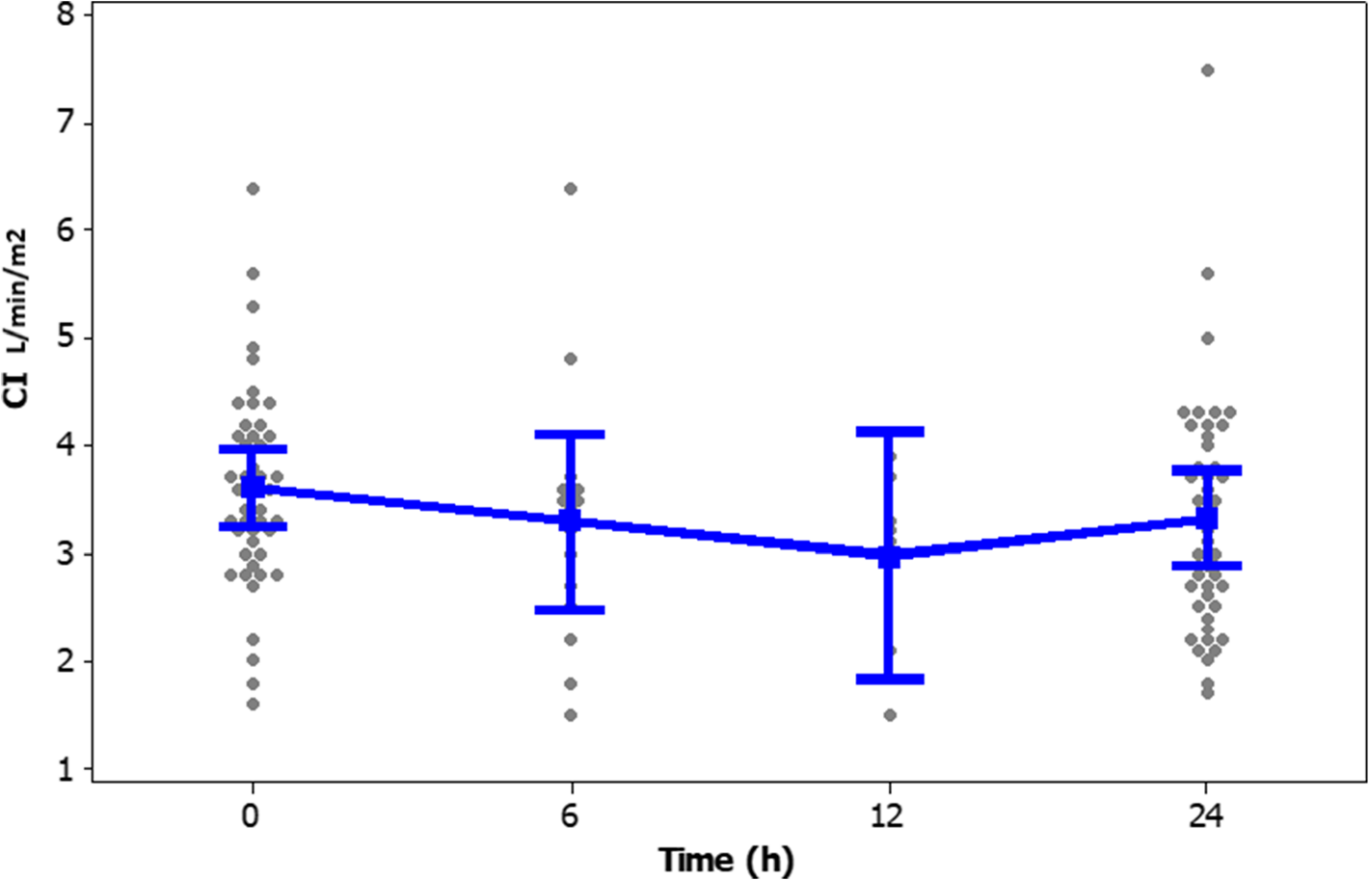
Mean and Bonferroni 95% CI of CI during 24-h of follow-up

**Fig. 7:**
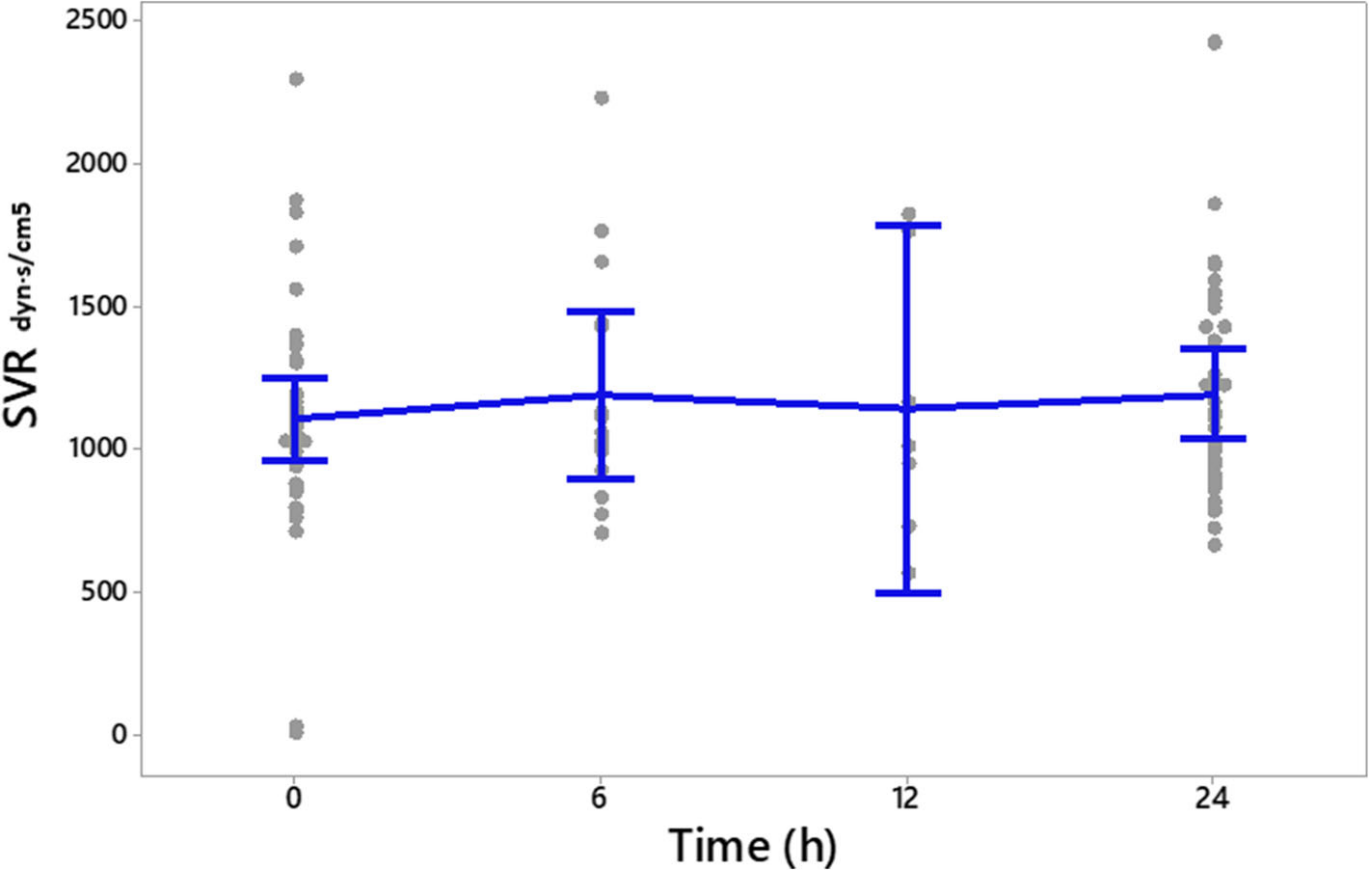
Mean and Bonferroni 95% CI of SVR during 24-h of follow-up

The frequency (%) of different heart rate ranges in patients at the time of enrollment and after 24-h of the study period is presented in Fig. 2. The frequency (%) of different ranges of SVI in patients at enrollment and after study period presented in Fig. 3. Clinical outcomes of amiodarone-treated patients presented in Table 2. Figures 4, 5, 6, 7 illustrates Mean and Bonferroni 95% CI of HR, MAP, CI, and SVR during 24-h of follow-up. During amiodarone infusion, the heart rate, mean arterial pressure, cardiac index, norepinephrine infusion rate, and stroke volume index changed significantly between study initiation and 24-h follow-up. As displayed in Fig. 4, heart rate appreciably decreased from 121.0 (116.5140.0) at baseline to 91.5 (89.3108.0) at time of amiodarone discontinuation (*P* < 0.001) (Table 2). At the initiation of the study, SVR was 1088.6 ± 373.9 dynes/seconds/cm^− 5^, although not statistically significant, it was numerically higher at the end of the study period 1193.7 ± 403.5 dynes/seconds/cm^− 5^ (*P* = 0.109). A similar finding observed for serum lactate, with lactate falling from an initial 25.5(20.8,33.0) to 19.5(11.8,32.3) at the end of the study (*P* = 0.057). Mean and Bonferroni 95% CI of clinical outcomes during 24-h of follow-up presented in Figs. 4, 5, 6, 7.

**Table 2:** Clinical outcome of the patients

Variable	Amiodarone treated patients (***n = 46***)			
Outcome	Baseline	After 24 h	Difference	***P***-value
HR	121.0(116.5140.0)^b^	91.5(89.3108.0)		< 0.001^d^
MAP	98.4 ± 9.3 ^a^	89.1 ± 9.1	9.3 ± 8.9	< 0.001 ^c^
CI	3.66 ± 0.92 ^a^	3.29 ± 1.10	0.37 ± 0.95	0.013 ^c^
SVR	1088.6 ± 373.9 ^a^	1193.7 ± 403.5	− 105.0 ± 312.8	0.109 ^c^
SVI	31.7 ± 8.5 ^a^	33.8 ± 9.4	−2.1 ± 6.9	0.054 ^c^
NE	11.0(10.0,12.0) ^b^	9.0(8.0,12.0)		0.023 ^d^
LACTATE	25.5(20.8,33.0) ^b^	19.5(11.8,32.3)		0.057 ^d^

^a^ Mean ± SD, ^b^ Median (Q1, Q3), ^c^ paired samples t-test, ^d^ Wilcoxon’s signed-rank test

## Discussion

This prospective, single-arm preliminary study conducted on the septic patients with persistence tachycardia after being adequately resuscitated. This is the first study of amiodarone for the management of tachycardia in this population. Our results showed that amiodarone successfully reduced the heart rate. The patients had improved hemodynamic state as indicated by an increase in cardiac index and SVI. The drug was well tolerated. Amiodarone did not increase the need for vasopressor and none of the patients experienced episodes of refractory hypotension.

The septic shock patients predispose to tachycardia, even after correcting other causes of tachycardia [15]. This tachycardia is due to sympathetic overstimulation. Tachycardia worsens the outcome of the septic shock patients by increasing myocardial workload and oxygen demand. The time of diastolic relaxation is shortened, the perfusion of the coronary artery diminished, which in turn contributes to diastolic dysfunction and worsening of myocardial performance [8]. The results of the present study indicated that amiodarone could effectively control heart rate in the septic patients with tachycardia.

In this study we presumed that amiodarone is able to improve diastolic dysfunction due to its pharmacological properties and the aim of the study was to evaluate the efficacy of amiodarone in sepsis-induced tachycardia following calcium dysregulation and sepsis. Therefore, we excluded AF patients, since the hemodynamics of a patient with concurrent septic shock and AF are very unstable [31]. If the AF patient is converted, the cardiac output is increased in this patient [31]. While this improvement in hemodynamic parameters cannot be differentiated whether it is due to rhythm correction and atrial and ventricular synchrony, or due to improvement in diastolic dysfunction induced by sepsis and septic shock. Another concern is that the technique applied in this study for measurement of hemodynamic parameters, USCOM, does not have the required accuracy in AF patient [32]. In these conditions, the stroke volume waves are irregular, and the diastolic pressure filling becomes large and small. In fact, in AF patients, stroke volume is not dependent on the strength of myocytes, but is caused by the cardiac chamber not being full. Thus, the hemodynamic outcomes measured in this study, namely cardiac output and stroke volume, were all affected by AF as a confounding factor. So we exclude AF patients from our study.

During the septic shock, the release of catecholamines increases to maintain systemic vascular resistance and blood pressure. However, high circulating catecholamines have cardiomyotoxicity effects via oxidative injury, inflammation, cellular apoptosis and necrosis in the myocardium [33]. Furthermore, the duration of exposure to catecholamines and their cumulative dosage contribute to poor neurological outcomes and hemodynamic state [14]. Therefore, if a drug could reduce the demand for vasopressor, it would have a potential role in improving the outcomes of these patients. As indicated in this study, amiodarone did not increase the demand for exogenous vasopressor and hemodynamic instability. This might be due to an increase in SVR.

The ideal threshold for heart rate is difficult to define. It depends on systemic hemodynamic status, comorbidities and individual characteristics. In the current study, we considered the heart rate ranging from 80 to 94 PBP as a sufficient therapeutic threshold [17]. Although in this study, only 56.52% of patients reached the predefined heart rate threshold, the most outstanding achievement of using amiodarone was the improvement of patients’ hemodynamic status, despite not reaching this target. As mentioned, the primary outcome assessed in this study was heart rate control in the septic shock patients. The results of this study showed that amiodarone could significantly reduce heart rate. When evaluating the secondary outcomes, it was found that patients had lower MAP by 9.30 mmHg ±8.95, which does not seem a positive finding at first glance. However, consideration should be taken that the baseline MAP of the patients was higher than the target (MAP> 65 mmHg), which indicates overuse of NEP. During the study, no hypotension episode was observed and amiodarone did not increase the need for a vasopressor. Moreover, this drug improved hemodynamic parameters as indicated with enhanced CI and SVI. This target threshold has previously shown to achieve in all the patients receiving esmolol than the control group [17]. Perhaps a possible explanation for this finding is that esmolol, unlike amiodarone, has an ultrashort onset of action, resulting in faster drug up-titration. We might have achieved similar results if we had extended the duration of the study or instead of defining a fixed-dose amiodarone protocol, we were able to increase the rate of amiodarone infusion.

In previous studies, β-blockers have successfully used to control heart rate in the septic shock. In the septic shock patients receiving vasopressor, Esmolol infusion improved hemodynamic and clinical outcomes [16, 17]. Although esmolol is a potential medication to reduce the heart rate, amiodarone has a long history of use in patients with heart failure. Amiodarone maybe an appropriate potential in patients with sepsis and cardiogenic shock with cardiac index ≤2.5, which calls for future studies in this subpopulation.

In the present study, the most important advantage of using amiodarone, along with its heart rate- controlling, was improving the patient’s hemodynamic condition by increasing cardiac index, stroke volume and better systemic vascular resistance, and no need for vasopressor demand. This leads to improvement in ventricular filling during diastole, and consequently an increase in cardiac performance, stroke volume, and organ oxygenation, while protecting against the destructive effects of catecholamines. As stated, amiodarone remarkably decreased lactate level, indicating an enhancement in tissue perfusion and organ function.

### Study limitation and future prospects

Although the findings of this preliminary study were promising, these results need to be interpreted with caution and confirmed in a larger group of the septic shock patients with tachycardia. The major limitation of the present research was the relatively short duration of study and not following up the patients to report their eventual outcome and survival. Another limitation of this study was adopting a heart rate threshold and not individualizing the protocol based on the patients’ characteristics. Third, based on the present study, we cannot conclude that the beneficial effects observed by the drug were solely due to controlling heart rate or due to the positive impacts of amiodarone on calcium dysregulation, etc. The last limitation is that in our medical center, the patient’s tachycardia has been controlled and modified for many years, and the use of amiodarone is a routine practice. As a result, we did not find it ethical to leave tachycardia untreated in these patients. Therefore, this study designed as “before and after”, and not as a “clinical trial”, with a control arm.

We recommend that future studies expanded to include amiodarone as a new practice in medical centers, which do not routinely manage tachycardia. It is suggested to evaluate different infusion rates of this medication to achieve the optimal dosage in this group of patients to control heart rate and also extent the duration of the study to be able to conclude whether amiodarone could also improve survival in this population or not. Investigation of the effects of amiodarone at the level of cellular level will help to address this point.

## Conclusion

The results of this prospective, single-arm preliminary study on the septic patients with persistent tachycardia indicated that amiodarone successfully reduced the heart rate. Moreover, the patients had improved hemodynamic state as indicated by an increase in cardiac index and SVI. The drug was well tolerated. Amiodarone did not increase the need for vasopressor and none of the patients experienced episodes of refractory hypotension. The results of this study were promising, suggesting the use of amiodarone for the management of persistent tachycardia in the septic patients.

## Data Availability

The datasets used and/or analysed during the current study available from the corresponding author on reasonable request. The raw SPSS file of this study before analysis is available upon your request.

## Abbreviations

ICU: intensive care unit
SV: stroke volume
SVR: systemic vascular resistance
MAP: mean arterial blood pressure
CO: cardiac output
SVI: Stroke Volume Index
CI: cardiac index
USCOM: Ultrasonic Cardiac Output Monitor
ECMO: Extracorporeal membrane oxygenation
ARDS: Acute respiratory distress syndrome
PaO_2_/FiO_2_: arterial oxygen partial pressure to fractional inspired oxygen
FTc: flow time corrected
PLR: passive leg raising
BPM: beats per minute
SOFA: sequential organ failure assessment
GCS: Glasgow Coma Scale
RASS: Richmond Agitation Sedation Scale
SD: standard deviation
Cis: confidence intervals
NE: Norepinephrine
AF: Atrial fibrillation
